# Use of outpatient medical care by headache patients in Germany: a population-based cross-sectional study

**DOI:** 10.1186/s10194-020-01099-1

**Published:** 2020-05-11

**Authors:** Britta Müller, Thomas Dresler, Charly Gaul, Tim Jürgens, Peter Kropp, Anna Rehfeld, Olaf Reis, Ruth Ruscheweyh, Andreas Straube, Stefanie Förderreuther

**Affiliations:** 1 Institute of Medical Psychology and Medical Sociology, University Medicine Rostock, Gehlsheimer Str. 20, D-18147 Rostock, Germany; 2 Department of Psychiatry and Psychotherapy, University Hospital Tübingen, Tübingen, Germany; 3 LEAD Graduate School & Research Network, University of Tübingen, Tübingen, Germany; 4 Migraine and Headache Clinic Königstein, Königstein, Germany; 5 Department of Neurology, University Medicine Center Rostock, Rostock, Germany; 6 Clinic for Child and Adolescent Psychiatry, University Medicine Rostock, Rostock, Germany; 7 Department of Neurology, Ludwig Maximilian University of Munich, Munich, Germany

**Keywords:** Headache, Population-based study, Headache-specific physician consultation, Patients’ occupational status

## Abstract

**Background:**

Headache sufferers in need of professional health care often do not utilize the care available, and factors influencing headache-specific physician consultation are not yet understood. Objectives of this study are (1) to assess self-reported headache-specific physician consultations and (2) to identify headache-related and sociodemographic predictors.

**Methods:**

Data of a random sample of the general population in Germany aged ≥14 years were analyzed (*N* = 2461). A multivariate binary logistic regression was conducted to identify a parsimonious model to predict physician consultation.

**Results:**

50.7% of the participants with headache reported at least one headache-specific physician consultation during lifetime. Of these, 53.6% had seen one, 26.1% two, and 20.3% more than two physicians because of their headaches. The odds of physician consultation increased with the number of headache days per month (HDM) [(reference HDM < 1) HDM 1–3 (*OR* = 2.29), HDM 4–14 (*OR* = 2.41), and HDM ≥15 (*OR* = 4.83)] and increasing Headache Impact Test score (HIT-6) [(reference “no or little impact”) moderate impact (*OR* = 1.74), substantial impact (*OR* = 3.01), and severe impact (*OR* = 5.08)]. Middle-aged participants were more likely to have consulted than younger and older ones [(reference 14–34 years) 35–54 years (*OR* = 1.90), 55–74 years (*OR* = 1.96), ≥75 years (*OR* = 1.02)]. The odds of physician consultation among self-employed subjects were lower than among employed manual workers (*OR* = 0.48). The living environment (rural versus urban) did not have an influence on the consultation frequency.

**Conclusion:**

The results indicate that apart from burden-related factors (headache frequency; headache impact), health care utilization patterns are also influenced by patients’ occupational status and age. Further research is needed to analyze whether the lower consultation rate means that the self-employed have a higher risk of chronification or that they have more effective self-management strategies regarding headache.

## Introduction

**Fig. 1:**
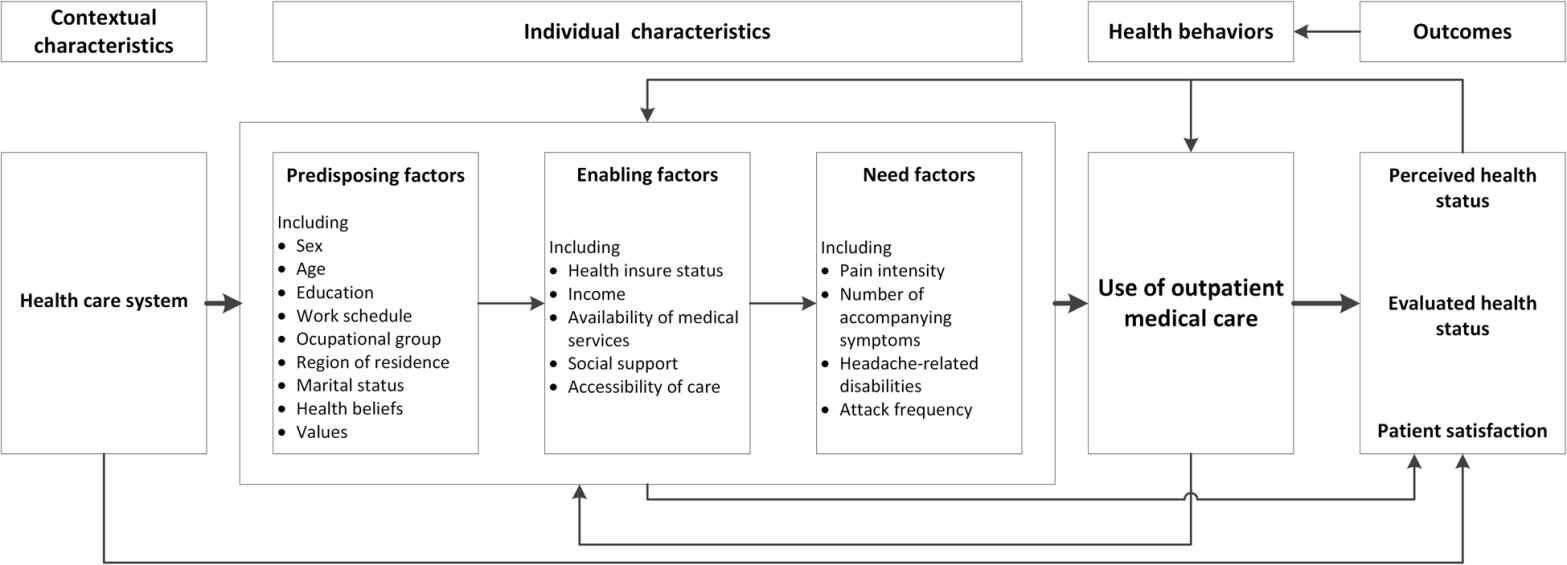
Revised version of the Behavioral Model of Health Services Use: modified for persons with headache [5, 6]

The use of outpatient medical care by headache sufferers is an essential prerequisite for correct diagnosis and initiation of effective treatment [1]. It is estimated that in Europe only about 50% of headache sufferers in need of professional health care (including general primary care, headache specialists or specialized headache centers) actually receive it [2]. Health services research has shown that, apart from need factors, health care utilization is also dependent on individual and contextual determinants [3]. A theoretical framework for these determinants is provided by the Behavioral Model of Health Services Use, developed by Andersen and Newman [4]. The core of this model contains three components: (1) predisposing factors (sociodemographic characteristics such as sex, age, education, work schedule, occupational group, region of residence, marital status, health beliefs, values), (2) enabling factors (resources that a person directly accesses in order to use health care, e.g. health insurance status, income, availability of medical services, social support, accessibility of care), and (3) need factors (perceived need for health care; evaluated need by professional assessments). In its fourth revision, the model was extended by two additional factors: (4) outcome (perceived and evaluated health status after care utilization, satisfaction with medical treatment), and (5) health care system. The revised version is also based on the assumption of feedback loops between outcome on the one hand and predisposing, enabling, need factors and health service care utilization on the other [5, 6] (see Fig. 1).

Headache research provides empirical evidence that some of the need factors are significantly associated with consulting behavior: 1) pain intensitiy [7–16], 2) number of accompanying symptoms [8, 10, 14], 3) disabilities from headache [7, 14, 17, 18], and 4) attack frequency [7, 13, 16, 18, 19]. The higher the burden of suffering, the higher the likelihood of consulting a physician. However, only little is known about predisposing and enabling factors regarding people with headache. In their review, Hunt and colleagues [20] considered 11 studies with a focus on the association between sex and help-seeking for headache. They found that five studies reported a greater consultation rate for women than men, whereas no study found a greater consultation rate for men. However, summarizing their review, the authors state that the findings were inconsistent and the studies had considerable limitations (e.g., plurality of the definitions “headache” and “consulting”, different periods of time, odds ratios adjusted for different variables). Regarding age, two studies showed an increase in physician consultation with increasing age [10, 14], two studies reported a decrease with age [11, 18], and one study reported age-independence [8]. The findings on the influence of marital status are also inconsistent [13, 14, 17]. Two studies from Asia reported higher consultation rates in rural as compared to urban areas [10, 17]. The empirical evidence for education as predictor is low [10, 11]. Professional characteristics have hardly been investigated so far. Only Lipton and colleagues [8], using data from the 2009 American Migraine Prevalence and Prevention study, found no difference in the consultation behavior of migraine sufferers employed full-time or employed part-time.

Overall, it appears that potential effects of predisposing and enabling factors to treatment-seeking behavior due to headache in general are still largley unknown. It should be noted that about half of the studies investigated US American samples [8, 13, 14, 16, 21–26]; in Europe, treatment-seeking behavior among headache sufferers has been researched mainly in the UK [18, 27, 28] and France [7, 9, 29, 30]. For Germany, there are only two studies [12, 31]. Neither predisposing nor enabling factors were analyzed in these studies.

The objectives of our study are (1) to assess self-reported headache-specific physician consultations in a large, representative, population-based German sample aged ≥14 years and (2) to to identify predictors that, in addition to need factors, are associated with headache-specific physician consultation.

## Methods

### Participants

A random general population sample in Germany with participants aged 14–94 years was investigated using a cross-sectional questionnaire survey with face-to-face contacts. Of a total of 4838 persons selected, 2510 took part in the study (52%). Methods and reasons for non-participation were detailed previously [32]. A weighted random sample was created based on an adjustment factor, the structure of which regarding age, sex, household size, and population by federal state corresponds to that of the German population. Evaluable data were available from 2478 participants [32]. From the current analysis, 17 subjects were excluded due to missing answers regarding physician consultation. Thus, *n* = 2461 participants (weighted sample *n* = 2459) were entered into the present analysis. Data were collected from September to November 2016. All participants gave their written informed consent. The Ethics Committee of the Faculty of Medicine, University of Leipzig, reviewed and approved the study (297/16-ek).

### Questionnaires

In the present study, the standardized questionnaire about headache and headache treatment started with the screening question “Did you have a headache during the last 6 months?” Participants answering in the affirmative were asked if they did ever consulted a physician (or more than one) because of their headaches (yes/no). In this way the lifetime physician consultation could be determined. If the answer was affirmative, participants were asked to specify the number of physicians consulted. Furthermore, participants were asked to rate their headache frequency on a five-point ordinal scale: (1) < 1 day per month; (2) 1–3 days per month; (3) 4–14 days per month; (4) ≥ 15 days per month, but not daily; (5) daily. For statistical analysis, the five categories were summarized into four classes of headache days per month (HDM): < 1 day per month, 1–3 days per month, 4–14 days per month, ≥ 15 days per month. The impact of headache on daily life was assessed using the German version of the Headache Impact Test (HIT-6) [33]. The HIT-6 is a six-item, self-administered questionnaire with three items assessing the impact of headache during the past 4 weeks and three items without a specific time frame. Five response categories were given: “never”, “rarely”, “sometimes”, “very often”, and “always”. The total score can range from 36 to 78. Higher scores indicate a greater impact of headaches on the ability to function on the job, at school, at home and in social situations. The HIT-6 grades four levels of headache impact: little or no impact (< 50), moderate impact (50-55), substantial impact (56–59) and severe impact (> 59).

To identify potential predisposing and enabling predictors of headache-specific physician consultation, a standardized sociodemographic questionnaire was used to assess the following parameters: age, sex, school education, work schedule, type of occupation and composition of household. School education was summarized into three classes: lower school education (school-leaving qualification, lower secondary school qualification, secondary school qualification), higher school education (qualification for university entrance), still in education. Based on the work schedule categories, four classes were formed: full-time (≥35 h per week), part-time (< 35 h per week), pensioners, other (volunteers; temporarily absent from work due to maternity or parental leave, unemployed, homemaker, in the education or training process). Based on the Erikson–Goldthorpe–Portocarero schema, the occupational groups were arranged in 18 categories and afterwards summarized into four classes: employed manual workers, non-manual employees and officials, self-employed persons, and others who had never had a job [34]. According to Goldthorpe, employed manual workers have entered into a traditional employment relationship in which work is performed in exchange for wages [35]. Employed manual workers not only include unskilled and semi-skilled workers, but also qualified skilled workers, who are highly trained and bear corresponding responsibilities. Non-manual employees perform predominantly commercial, higher technical, office or managerial activities. Officials are appointed, employed, and removed by the Public Sector Service and Loyalty Law. Officials can range from ordinary civil service to higher civil service. The self-employed can be divided into freelancers and tradesmen. Generally, tradesmen are essentially free to organise their activities and determine their working hours, and they are not bound by supervision. In contrast to employed manual workers, non-manual employees, and officials, self-employed persons are those who are not in an employment relationship with an employer or company. Persons who never had a job are homemakers or participants still in school education, vocational training or at university. Another potential predictor, the residential environment, was classified into rural and urban areas based on the sampling plan. A rural region was defined as less than 20,000 inhabitants living in a community that was neither close to large cities nor part of a city-region or metropolitan area [36].

### Statistical analysis

Two-tailed tests (Fisher’s exact test, Welch’s *t* test, Pearson’s χ^*2*^ test) were used to test for differences between participants with and without headache-specific physician consultation. Multiple comparisons were adjusted using the Holm-Bonferroni procedure [37]. This method is a sequential approach to increasing the power of statistical tests while keeping under control the familywise type 1 error rate. The Holm-Bonferroni test is more powerful than the simple Bonferroni correction and it is recommended to be used by Schochet [38]. The recommendations by Agresti and Kateri [39] were applied to analyze categorical variables. They suggest using adjusted standardized residuals (standardized Pearson residual) to evaluate the deviations of observed and estimated expected frequencies. An adjusted residual exceeding 2 or 3 in absolute value indicates a rather unlikely deviation which can be interpreted as significant. In the present analysis, deviations exceeding a value of 2 were considered significant. In a second step, a multivariate binary logistic regression was conducted to identify a parsimonious model to predict physician consultation (yes/no). All variables associated with headache-specific physician consultation (indicated by a *p* value ≤.05) were simultaneously entered as predictors in the equation. All statistical analyses were carried out with the weighted data set and were performed using IBM SPSS Statistics 24.

## Results

The weighted 6-month prevalence of self-reporting headache amounted to 38.6%. Of the participants with headache, 50.7% reported having consulted a physician during lifetime because of their headaches. Of these, 53.6% consulted one, 26.1% two, and 20.3% more than two physicians.

**Fig. 2:**
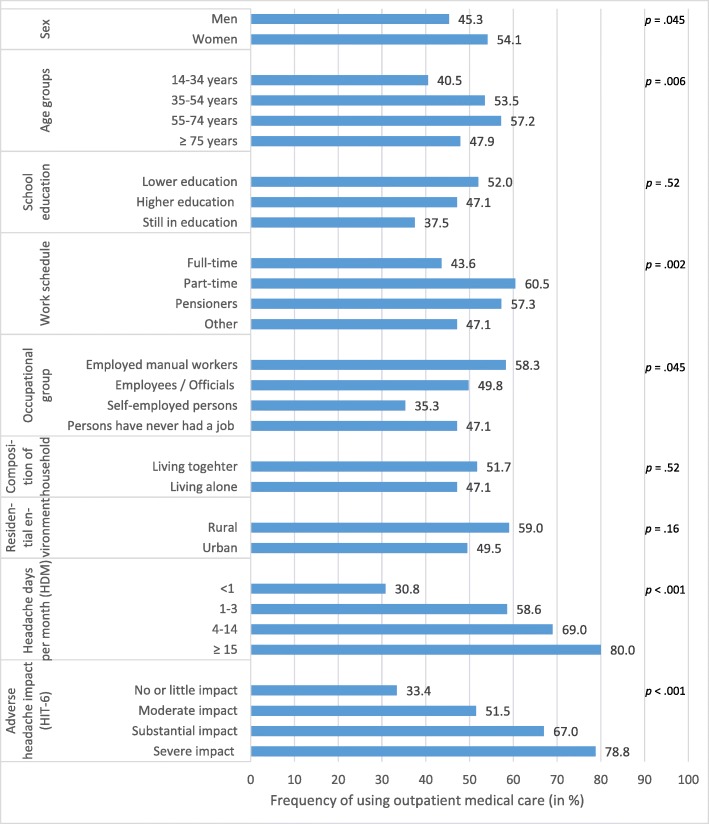
Frequency (in %) of using outpatient medical care among subjects reporting headache (weighted random sample; adjusted *p* values based on the Holm-Bonferroni method)

Table 1 shows the sociodemographic and health-related characteristics of consulting and non-consulting subjects with headache. Additionally, Fig. 2 shows the frequency of using outpatient medical care among subjects reporting headache. Women sought medical advice for their headache significantly more often than men. Participants aged 14–34 years were under-represented (adjusted residual: − 3.8) and those aged 55–74 years were over-represented (adjusted residual: 2.5) among consulters. There was also a significant association between consultation behavior and work schedule. It was observed that those with a part-time schedule (adjusted residual: 2.9) and pensioners (adjusted residual: 2.3) were consulting significantly more than expected, while participants with a full-time schedule (adjusted residual: − 3.5) were consulting significantly less than expected. Furthermore, there was a significant association between consultation behavior and occupational group: self-employed persons were less likely to consult a physician (adjusted residual: − 2.6) and employed manual workers were over-represented (adjusted residual: 2.5). Employed manual workers and self-employed persons did not differ in the 6-month prevalence of migraine, tension type headache, cluster headache, and other headache (data can be requested from the first author). Moreover, a significant association was found between consultation behavior and frequency of headache. This effect was due to the smaller than expected numbers of participants with HDM < 1 (adjusted residual: − 9.6) among consulters and the increased numbers of participants with HDM 1–3 (adjusted residual: 4.2), HDM 4–14 (adjusted residual: 4.8), and HDM ≥15 (adjusted residual: 4.0). Finally, patients consulting a physician reported a higher headache impact compared to non-consulters: Participants with no or little impact (HIT-6 scores: < 50) were under-represented (adjusted residual: − 9.4) and those with substantial impact (HIT-6 scores: 56–59) (adjusted residual: 3.5) and severe impact (HIT-6 scores: > 59) (adjusted residual: 8.6) were over-represented. No differences were found for school education, composition of household, and living environment.

**Table 1:** Sociodemographic and health-related characteristics of the study sample

Variable		Total sample *(N* = 949)	Physician consultation		*p* value (adj.)^d^
			Yes (*N* = 481)	No (*N* = 468)	
Sex, *n* (%)	Men	358 (37.7)	162 (33.6)^c^	196 (41.9)	.045^a^
	Women	592 (62.3)	320 (66.4)^c^	272 (58.1)	
	(missings *n* = 0)				
Age group, *n* (%)	14–34 years	252 (26.6)	102 (21.2)	150 (32.1)	.006^b^
	35–54 years	355 (37.4)	190 (39.5)	165 (35.3)	
	55–74 years	269 (28.3)	154 (32.0)	115 (24.6)	
	≥75 years	73 (7.7)	35 (7.3)	38 (8.1)	
	(missings *n* = 0)				
School education, *n* (%)	Lower education	725 (76.6)	377 (78.5)	348 (74.5)	.52^b^
	Higher education	206 (21.8)	97 (20.2)	109 (23.3)	
	Still in school	16 (1.7)	6 (1.3)	10 (2.1)	
	(missings *n* = 3)				
Work schedule, *n* (%)	Full-time	376 (40.0)	164 (34.5)	212 (45.6)	.002^b^
	Part-time	177 (18.8)	107 (22.5)	70 (15.1)	
	Pensioners	218 (23.2)	125 (26.3)	93 (20.0)	
	Other	170 (18.1)	80 (16.8)	90 (19.4)	
	(missings *n* = 6)				
Occupational group, *n* (%)	Employed manual workers	192 (20.6)	112 (23.9)	80 (17.2)	.045^b^
	Employees / Officials	605 (64.8)	301 (64.2)	304 (65.5)	
	Self-employed persons	68 (7.3)	24 (5.1)	44 (9.5)	
	Persons who never had a job	68 (7.3)	32 (6.8)	36 (7.8)	
	(missings *n* = 12)				
Composition of household, *n* (%)	Living together	745 (78.5)	385 (80.0)	360 (76.9)	.52^a^
	Living alone	204 (21.5)	96 (20.0)	108 (23.1)	
	(missings *n* = 0)				
Residential environment, *n* (%)	Rural	122 (12.9)	72 (15.0)	50 (10.7)	.163^a^
	Urban	827 (87.1)	409 (85.0)	418 (89.3)	
	(missings *n* = 0)				
Headache days per month (HDM), *n* (%)	< 1	360 (37.9)	111 (23.1)	249 (53.2)	< .001^b^
	1–3	399 (42.0)	234 (48.6)	165 (35.3)	
	4–14	145 (15.3)	100 (20.8)	45 (9.6)	
	≥15	45 (4.7)	36 (7.5)	9 (1.9)	
	(missings *n* = 0)				
Adverse headache impact (HIT-6), *n* (%)	No or little impact	395 (42.4)	132 (27.6)	263 (57.9)	< .001^b^
	Moderate impact	235 (25.2)	121 (25.3)	114 (25.1)	
	Substantial impact	109 (11.7)	73 (15.3)	36 (7.9)	
	Severe impact	193 (20.7)	152 (31.8)	41 (9.0)	
	(missings *n* = 16)				

Weighted random sample; hrs/wk., hours per week

^a^ Fisher’s exact test

^b^ Pearson’s χ^*2*^test

^c^ Please note that the sum of men and women among consulters slightly deviates from the overall number because the weighted data

^d^ Adjusted *p* values based on the Holm-Bonferroni method

The logistic regression analysis revealed four predictors remaining significant in the final equation: headache frequency, headache impact, age, and occupation. Higher frequency of headache, a higher impact of headache, middle age and not being self-employed significantly increased the probability of a headache-specific physician consultation. The overall significance of the model was χ^2^ (16, *N* = 932) = 190.76, *p* < .001 (Likelihood-Ratio-Test), with one quarter of explained variance (Nagelkerke-*R*^2^ = 0.25). The detailed model is shown in Table 2.

**Table 2:** Factors associated with physician consultation: Logistic regression analysis

Variable (Reference)	*OR*	95% *CI*	*p* value
Headache days per month (HDM) (< 1^a^)			< .001
1–3	2.29	1.65–3.19	< .001
4–14	2.41	1.49–3.89	< .001
≥ 15	4.83	2.02–11.6	< .001
Adverse headache impact (HIT-6) (No or little impact^a^)			< .001
Moderate impact	1.74	1.22–2.48	.002
Substantial impact	3.01	1.84–4.92	< .001
Severe impact	5.08	3.21–8.04	< .001
Sex (Male^a^)			
Women	1.00	0.70–1.41	.98
Age group (14–34 years^a^)			.003
35–54 years	1.90	1.25–2.83	.002
55–74 years	1.96	1.19–3.24	.008
≥ 75 years	1.02	0.46–2.29	.96
Work schedule (Part-time^a^)			.20
Full-time	0.71	0.46–1.10	.13
Pensioners	1.00	0.56–1.79	.99
Other	0.60	0.34–1.06	.076
Occupational group (Employed manual workers^a^)			.033
Employees / Officials	0.69	0.46–1.03	.067
Self employed persons	0.48	0.24–0.92	.027
Persons who never had a job	1.40	0.65–2.99	.39

Weighted random sample; *OR* odds ratio, *CI* confidence interval

^a^reference; HIT-6, Headache Impact Test, range 6–78, higher score indicates a greater impact of headaches on the ability to function on the job, at school, at home and in social situations

## Discussion

The study analyzed the consultation behavior concerning headache in a sample of the general population. 39% of the population reported headaches in the last 6 months. Half of the headache-sufferers never consulted a physician because of their headache. Higher frequency of headache, a higher impact of headache, middle age and not being self-employed significantly increased the probability of a headache-specific physician consultation. Importantly, self-employed subjects with headache were less likely to consult a physician than employed manual workers with headache.

Given a headache prevalence of 39% in our study, about 20% of the German population aged 14 and older consulted a physician during lifetime for this reason. No other population-based studies with a comparable age range were identified that examined the lifetime consultation rate of headache sufferers. Regarding specific headache diagnoses, the literature indicates differences: For migraine, based on the higher burden, consultation rates are usually higher, ranging from 46% to 86% [7–10, 17, 23, 40–42] compared to tension-type headache (TTH) ranging from 16% to 45% [17, 40, 41, 43]. Regarding the Behavioral Model of Health Services Use, frequency and impact of headache were identified as two need factors, and age and occupation as two predisposing factors associated with headache-specific physician consultation.

The finding regarding the frequency of headache - the more days with headaches per month the greater the likelihood of consulting a physician - is well in line with the study by Wang et al. [10]. They reported a higher likelihood of physician consultation by headache sufferers with a headache frequency of 2 days per week compared to those with less than 1 day per week.

Additionally, the likelihood of a consultation increases with increasing headache impact (as measured by HIT-6). This association was also found in population-based studies conducted in France [7], China [17] and Germany [12]. We assume that headache impact is influenced by the duration of attacks, their intensity and the numbers of symptoms. Furthermore, the negative or positive reactions headache sufferers experience from their social environment may moderate the association between headache and headache impact [44].

The finding that consultation was more likely with increasing age, particularly up to 74 years, is consistent with other studies regarding both lifetime consultation [14] and consultation within the last 12 months [10]. One likely reason is the increasing frequency of migraine attacks with age and therefore also an increase in chronic migraine [45, 46]. Furthermore, since the lifetime prevalence of medical consultations for headache was collected, it is rather trivial that the overall consultation rate increased with age. However, it has been shown that participants older than 74 years of age consulted a physician less frequently than participants aged 55-74 years. Besides a possible recall bias, this could be due to a cohort effect: it is possible that older cohorts had less awareness of headache treatment than younger cohorts, or headache may have a lower priority in the older cohort than in younger ones.

Somewhat surprising is that the consultation rate in rural areas were not lower than in urban areas, although there is a public discussion in Germany in the recent years about the deficit in medical care in rural areas. In regard of the frequency of consultation for headache seems to be evident deficit.

Lastly, our study revealed a novel finding regarding the occupational status. Compared to employed manual workers, self-employed people were less likely to utilize medical care regarding their headaches. This result is consistent with the findings of Stephan and Roesler [47]. For German self-employed people, the authors reported fewer physician consultations and fewer sick days compared to employees. From our point of view, there are two possible explanations for the lower physician consultation among the self-employed. I) Self-employed persons may be less willing than other occupational groups to take the sick role. Self-employed persons often report higher work motivation and higher work load, including longer and more irregular working hours compared to regular employees, as well as an “always on” work culture [47–50]. We assume that some stressors typically associated with self-employment may explain our findings, such as hours worked per week, business responsibilities, stronger dependency on clients, fewer options to delegate work and the need to avoid loss of earnings. These are occupational risks known to reduce the likelihood of utilizing outpatient medical care [48]. II) Furthermore, we assume structural variables as provided by the German health care system to influence health behavior. In Germany, it is required by law to have some form of health coverage, whether statutory or private. Every German has to pay health insurance in person in one way or another, which applies to self-employed persons, too. At the time of our survey 2016, about 87% of the Germans were covered by the statutory health insurance, 11% were privately insured, and 2% were covered by the state [51]. While almost all employed manual workers are covered by statutory health insurance, this only affects about 56% of the self-employed [52]. We identified several reasons, why a physician consultation might have negative consequences for self-employed persons, which do not occur with employed manual workers. First, employed manual workers on sick leave have the right to claim daily sickness benefit paid by their employer from the first day of illness until the sixth week of illness. However, self-employed persons covered by the statutory health insurance are not entitled to sickness benefit in their first 6 weeks of illness. They only receive sickness benefit from the health insurance from the seventh week of illness. We assume that these differences between the statutory insurance of employed manual workers and self-employed persons may result in different consultation behavior. The risk of loss of earnings during sick leave is only covered for manual workers although self-employed persons have a higher financial burden than employed workers. Second, 44% of self-employed persons are privately insured. That means, that non-use of insurances is more likely to be rewarded, compared to statutory insurances.

With recourse to the rational choice theory, we suspect that the lower number of consultants among self-employed persons is also due to the fact that self-employed persons estimate the cost-benefit relation of consulting a physician for a headache differently. Their benefits (diagnosis, effective treatment, symptom relief, medical advice) might be less likely to match their costs (loss of earnings during the consultation; loss of earnings during sick leave, cost of the treatment) when compared to employed manual workers [53].

### Strengths and limitations

The present study has several strengths. First, the analysis stems from current data on the utilization of outpatient medical care from a respresentative German sample. Second, our sample covered a wide age range including older and old participants. As a result, we were able to show an inverted U-shaped association between age and physician consultation. Third, unlike in a majority of previous studies, we have considered not only need-related, but also predisposing factors as potential predictors of physician consultation. We are the first who were able to show that consultation patterns are also influenced by patients’ occupational status.

However, several limitations apply. First, only retrospective self-reported data regarding physician consultation were analyzed, which can be influenced by recall bias. Second, we have no indications of the quality of the health advice during the consultations. Third, unlike the question about physician consultation, which concerned a lifetime period, the question about occupational group related to the time of data collection. Therefore, it cannot be excluded that the occupational group is not the same at the time of the consultation and the time of the data collection. Fourth, results obtained from a German sample may not be generalizeable to other countries and different health care systems. In Germany, there are office-based general practitioners and specialists working in private practice. About 87% of the German population have statutory health insurance and can freely choose their physicians within the statutory health-insurance system without prior referral [51]. It is to be expected that consultation rates will be significantly lower in countries with a higher proportion of privately insured or uninsured persons [3]. Fifth, low health care utilization of self-employed persons could also be the result of an over-utilization by manual workers seeking sick leave or there is an different motivational status of the self-employed. Sixth, in our analysis we did not differentiate between types of headaches (e.g. migraine, TTH). Seventh, the consultation behavior may also be influenced by the occurrence of comorbid disorders. Unfortunately no data were available to prove this hypothesis. Eighth, self-reports did not distinguish between different healthcare providers, e.g. general practitioners or emergency departments. Although it is known that headache is a common reason to visit the emergency department, little is known about sociodemographic and health-related characteristics of headache sufferers using an emergency department [54, 55]. Last, the group of self-employed persons is in itself heterogeneous regarding income, professional qualification, inherent business risks and the number of employees.

## Conclusion

The 6-month prevalence of self-reported headache in the German population is 39%. Only half of the headache sufferers ever consult a physician because of their headache. In addition to the expected need factors (frequency and impact of headache), the occupational status also impacted the consultation behavior. However, the reasons for the lower use of outpatient medical care among self-employed persons remain unclear. More research is needed to analyze whether self-employed persons have a higher risk of chronification or medication overuse headache (MOH) or whether they have more effective self-management strategies regarding headache. Physicians, in particular general practitioners, should be aware of the different headache-related consultation rates among the occupational groups. Non-headache related consultations of self-employed people could be used as an opportunity to assess the individual risk of chronification and MOH.

## Data Availability

The dataset generated and analyzed during this study is available from the corresponding author on reasonable request.

## Abbreviations

HDM: Headache days per month
HIT-6: Headache Impact Test
MOH: Medication overuse headache
TTH: Tension-type headache
